# Massive Augenschmerzen beidseits – Kurioser Fall in Zeiten der Corona-Pandemie

**DOI:** 10.1007/s00347-020-01201-7

**Published:** 2020-07-23

**Authors:** Kristin Röper, Chris Patrick Lohmann, Ines Lanzl

**Affiliations:** Klinikum rechts der Isar, Klinik und Poliklinik für Augenheilkunde, Technische Universität München, Ismaninger Str. 22, 81675 München, Deutschland

## Anamnese

Zwei männliche Patienten (35 Jahre und 22 Jahre) stellten sich nachts mittels Rettungswagen in der Notaufnahme einer chirurgischen Kreisklinik im Umland vor. Sie gaben beide seit dem späten Nachmittag massive Schmerzen an beiden Augen an. Der diensthabenden Chirurgin fielen ein deutlicher Abwehrspasmus, Lichtscheu und beidseits stark tränende Augen auf. Systemische Erkrankungen wurden verneint. Beide Patienten gaben massive Schmerzen im Augenbereich (visuelle Analogskala 8/10) an. Da sich diese auch unter der Therapie mit Metamizol 1 g intravenös (Sanofi-Aventis Deutschland GmbH, Frankfurt/Main) und unter oberflächlicher Kühlung nicht besserten, sondern sich sogar progredient zeigten, beschloss die chirurgische Kollegin, die Patienten mit dem Rettungswagen in die 80 km entfernte Universitäts-Augenklinik zu überweisen. Hier trafen die Patienten um 03.00 Uhr morgens ein.

## Klinischer Befund

Bei der Erstuntersuchung gaben die Patienten an, morgens gegen 10.30 Uhr am ersten Tag nach der Öffnung von Dienstleistungsbetrieben nach dem Corona-Lockdown beide den gleichen Friseur besucht zu haben.

Der klinisch schwerer betroffene Patient trage in der Nacht orthokeratologische Kontaktlinsen bei geringer Myopie, sonst lägen keine Augenvorerkrankungen vor und es waren keine Augenoperationen in der Vorgeschichte durchgeführt worden. Allergien wurden nicht genannt. Die Medikamentenanamnese war leer. Die Schmerzen hätten am späten Nachmittag angefangen.

**Figure 1:**
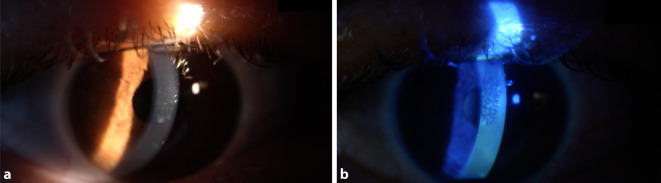

Bei dem klinisch schwerer betroffenen Patienten (35 Jahre) lag der bestkorrigierte Visus bei 0,8 beidseits. Bei dem klinisch leichter betroffenen Patienten (22 Jahre) lag der bestkorrigierte Visus bei 0,9 beidseits. Die Tensio war jeweils normoton. Im vorderen Augenabschnitt zeigten sich am rechten und am linken Auge jeweils eine Lidschwellung, eine injizierte Bindehaut, eine Hornhaut mit epithelialer, Fluoreszein-positiver Stippung (Abb. 1a, b). Intraokular zeigte sich ein reizfreies Auge. Fundusdetails erwiesen sich korneal bedingt als reduziert einsehbar, aber grob regelrecht.

Nach Installation von Proparakain Augentropfen (Ursapharm, Saarbrücken) zeigten sich beide Patienten komplett beschwerdefrei.

**Figure 2:**
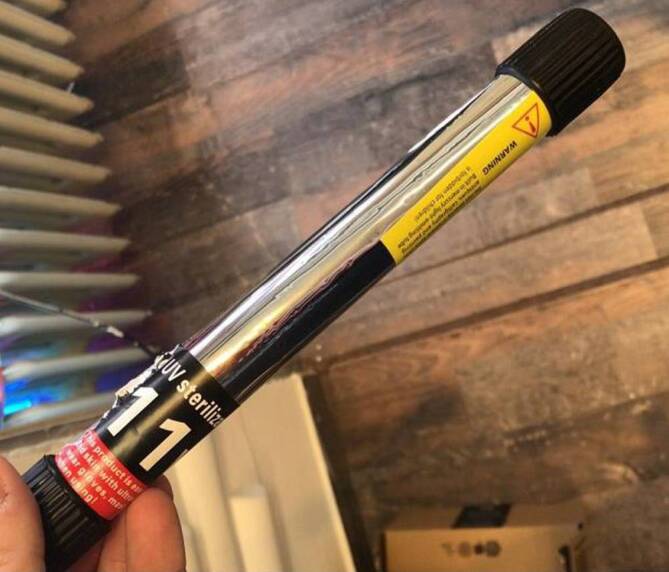

Auf Rückfrage gaben die Patienten an, dass der Friseur eine UV-Lampe im Friseursalon installiert hatte. Vonseiten der Bundesregierung war für die Öffnung nach dem Corona-Lockdown die Vorlage eines Hygienekonzepts erwünscht gewesen. Daher hatte der Friseur die UV-Lampe, die er normalerweise für die Reinigung seiner Gerätschaften benutze, an der Decke angebracht (Abb. 2).

## Wie lautet Ihre Diagnose?

## Therapie und Verlauf

Die Patienten wurden über die Diagnose einer sog. „Verblitzung“ (Keratitis photoelectrica) und die vermutete Pathogenese aufgeklärt. Wir begannen eine topische Therapie mit R/L Levofloxacin Augentropfen viermal täglich für 1 Woche (Santen GmbH, München) sowie eine intensive Therapie mit R/L Tränenersatzmittel. Für die weitere Analgesie erhielten die Patienten eine Bedarfsmedikation und wurden über die weitere voraussichtliche Symptomentwicklung aufgeklärt. Es wurde empfohlen, das Tragen der orthokeratologischen Kontaktlinsen bis zur vollständigen Abheilung zu pausieren. Auf den Friseur wurde zugegangen.

Bei der Verlaufskontrolle nach 72 h zeigte sich das Korneaepithel weiterhin mit kleineren Epitheldefekten, sodass wir zusätzlich eine Therapie mit Retinolpalmitat Augensalbe (Ursapharm, Saarbrücken) zur Nacht initiierten. Innerhalb kurzer Zeit zeigten sich die Patienten beschwerdefrei.

## Pathogenese, Hintergrund und Prävention

Die Hornhaut ist für das meiste Licht im sichtbaren Wellenlängenbereich durchlässig. Sie absorbiert jedoch Licht im ultravioletten (UV) Spektrum. Diese Absorption tritt in erster Linie im mehrschichtig unverhornten Plattenepithel der Kornea auf. Langfristige UV-Exposition hat am Auge pathologische Effekte (Pterygium, Katarakt, nichtmelanozytärer Hautkrebs, maligne Melanome) [1].

Im Falle einer Keratitis photoelectrica wurde die verträgliche Absorptionsdosis für das Korneaepithel überschritten, sodass es innerhalb von Stunden zu einem Zelltod und Desquamation kommt. Die starken Schmerzen entstehen dabei durch eine Freilegung des subepithelialen Nervenplexus. Die sensible Innervation der Kornea ist die dichteste des menschlichen Körpers. Sie ist etwa 20- bis 40-mal dichter als die Innervation des Zahnfleisches und etwa 300- bis 600-mal dichter als die Innervation der Haut [2, 3]. Anekdotisch wurde das Schmerzlevel einer Keratitis photoelectrica mit dem einer Geburt eines Kindes oder einer Nierenkolik verglichen.

Die Regeneration der Epitheloberfläche und die Besserung der Symptome treten innerhalb von 24–72 h auf. Studien an Tiermodellen zeigen, dass diese schnelle Reaktion auf den Makrophagen-Inhibitions-Faktor (MIF), einen integralen Bestandteil des Wundheilungssystems, zurückzuführen ist [4].

Aktuell wird eine intensive Therapie mit Tränenersatzmittel und suffizienter oraler Analgesie (falls notwendig bis hin zu wirksamen Opioiden der Stufe III im WHO-Stufenschema, z. B. Oxycodon) (Mundipharma GmbH, Frankfurt/Main) empfohlen. Eine prophylaktische topische antibiotische Abdeckung wird ebenfalls empfohlen. Eine Installation von zykloplegischen Tropfen oder eine Versorgung mittels Augensalbenverband entspricht nicht den aktuellen Empfehlungen [5]. Aufgrund der langfristigen Korneatoxizität sollte unter keinen Umständen eine Verschreibung von topischen Anästhetika erfolgen. Für eine initiale Diagnostik während der Erstuntersuchung dürfen diese verwendet werden [6].

Am 04.05.2020 durften Friseursalons im Bundesland Bayern nach dem Corona-Lockdown unter strengen Hygieneauflagen wieder öffnen. Insgesamt lässt sich der Vorfall unter einer möglichen Verunsicherung der Bevölkerung hinsichtlich von wirksamen Hygienemaßnahmen einordnen. Bei Nichteinhaltung der Hygienemaßnahmen drohten empfindliche Strafen. Ein möglicher Zusammenhang ist auch mit den erstaunlichen Vorschlägen des amtierenden US-Präsidenten Donald Trump zu sehen, der in einem Presseinterview am 23.04.2020 eine „UV-Therapie gegen Corona“ vorschlug: *„Angenommen, wir setzten den Körper einem ultravioletten oder einfach sehr starken Licht aus – und ich glaube, Sie [der Experte der Regierung] sagten, dass das noch nicht probiert wurde, aber ich glaube, Sie sagten, dass Sie das testen werden. Und dann sagte ich: Angenommen, man bringt das Licht in den Körper, was man machen kann, entweder durch die Haut oder auf einem anderen Weg – und ich glaube, Sie sagten, dass Sie das auch testen werden. Dann sehe ich noch die Desinfektionsmittel, die das Virus in einer Minute ausknocken. Und es gibt einen Weg, mit dem wir so etwas in der Art machen können – durch eine Injektion nach innen oder fast durch so etwas wie eine Säuberung. Es gelange so in die Lunge und zieht da eine enorme Nummer durch. Dafür braucht man Ärzte, aber es klingt interessant für mich.“* Später revidierte Präsident Trump seine Aussagen und betonte, sie seien sarkastischen Ursprungs gewesen [7].

**Diagnose:** R/L: massive Keratitis photoelectrica

Andere bekannte Ursachen für eine Keratitis photoelectrica sind sonst die sog. „Schweißerverblitzung“ und die sog. „Schneeblindheit“. Die beste Therapie der Keratitis photoelectrica ist die Prophylaxe. Patienten mit Exposition in der Freizeit sollten gut sitzende Sonnenbrillen nach EU-Norm verwenden. Besonders starker Schutz (z. B. für Expeditionen in Hochgebirgs- und Gletscherregionen) bietet die EU-Filterkategorie IV. Sonnenbrillen der Kategorie IV sind jedoch im Straßenverkehr nicht erlaubt und mit dem Symbol eines durchgestrichenen Autos gekennzeichnet. Personen mit potenzieller Exposition im Beruf sollten durch einen geeigneten Augen- und Gesichtsschutz geschützt werden [8].

## Fazit für die Praxis

- Die Keratitis photoelectrica ist ein akutes Erkrankungsbild, das starke Augenschmerzen verursacht, die sechs bis zwölf Stunden nach der Exposition gegenüber UV-Licht beginnen.
- Betroffene Patienten haben beidseits ein Fremdkörpergefühl, eine injizierte Bindehaut sowie oft ein leichtes Gesichtserythem und Lidödem. Eine Fluoreszeinfärbung kann punktuelle Hornhautfärbungen zeigen. Vereiterte Augen oder ein einseitiger Befall sprechen eher gegen diese Verdachtsdiagnose.
